# Residual-SwishNet: a deep learning-based approach for reliable lung cancer classification

**DOI:** 10.3389/fonc.2025.1729021

**Published:** 2026-01-26

**Authors:** Marriam Nawaz, Ali Javed, Abdul Khader Jilani Saudagar

**Affiliations:** 1 Department of Software Engineering, University of Engineering and Technology-Taxila, Taxila, Pakistan; 2 Information Systems Department, College of Computer and Information Sciences, Imam Mohammad Ibn Saud Islamic University (IMSIU), Riyadh, Saudi Arabia

**Keywords:** classification, deep learning, lung cancer, ResNet50, Swish

## Abstract

**Introduction:**

Lung cancer remains one of the primary causes of cancer-related deaths globally, emphasizing the urgent need for accurate and early diagnosis to improve patient outcomes. However, existing computer-aided detection systems often struggle with suboptimal feature extraction, low classification accuracy, and limited generalizability across datasets.

**Methods:**

To address these challenges, we propose a deep learning approach named Residual-SwishNet, explicitly designed for the lung cancer classification task. More specifically, we modified the ResNet50 framework by replacing the conventional ReLU activation function with Swish during the feature engineering phase. Further, we integrate three additional dense layers before the classification module to obtain an enriched feature representation. Lastly, we employ a Softmax output layer with Cross-Entropy Loss to tackle the class-imbalance issue.

**Results:**

The approach was rigorously evaluated on 2 publicly accessible datasets, named LUNA16 and IQOTH/NCCD, using precision, recall, F1-score, and accuracy as performance metrics. Experimental results demonstrate the superiority of our technique, achieving classification accuracies of 99.60% and 99.11% on the LUNA16 and IQ-OTH/NCCD datasets.

**Discussion:**

Our approach has significantly outperformed existing state-of-the-art techniques. These findings highlight the potential of the proposed model as a robust and reliable tool for lung cancer diagnosis.

## 1 Introduction

Lung cancer remains one of the most life-threatening health challenges worldwide, which originates from the abnormal and uncontrolled proliferation of cells within the lungs (1). This cancer is mainly categorized into 2 major categories: non-small cell lung cancer (NSCLC) and small cell lung cancer (SCLC) (2). Among these, NSCLCs are common and usually develop at a slower rate than SCLCs. Globally, lung cancer continues to be one of the most frequently identified tumors and a major cause of death. According to reports by the World Health Organization (WHO) and the International Agency for Research on Cancer (IARC), this category of cancer ranked as the second most diagnosed tumor in 2021, having 2.2 million new cases, exceeded only by breast cancer. Frighteningly, it was responsible for approximately 1.8 million deaths out of a total of 9.96 million cancer-related mortalities (3). The trend continued in 2022 and resulted in affecting nearly 2.5 million individuals and causing the same number of deaths, supporting its deadly impact (4). These alarming figures reflect the urgent need for more accurate, efficient, and early diagnostic techniques for this deadly disease. Timely recognition plays a pivotal part in refining prognosis and survival proportions. In this context, the current research is motivated by the persuasive challenge of high mortality and diagnostic complexity associated with lung cancer. This study proposes a novel deep learning (DL)-based method for recognizing lung cancer nodules with the aim of enhancing diagnostic reliability and supporting clinical decision-making. A crucial aspect of lung cancer analysis involves the detection and assessment of lung nodules, which are small, abnormal growths that appear on imaging modalities like chest X-rays and computed tomography (CT) scans (5). These nodules are either benign or malignant, with the latter potentially indicating early stages of lung cancer. Effective evaluation of these nodules is essential, as it can result in prompt intervention and significantly improved treatment outcomes. Consequently, routine screening, accurate classification, and conscientious monitoring of lung nodules form the cornerstone of modern strategies aimed at reducing the burden of lung cancer and improving patient care (6).

CT scans stand out as one of the most effective tools among the numerous imaging modalities used in the recognition of lung cancer. CT imaging provides a detailed cross-sectional representation of the chest, which permits clinicians to closely examine the lungs for signs of malignancy or other abnormalities (7). Its aptitude to produce high-quality, three-dimensional images promptly makes it a vital resource for the timely detection, recognition, and staging of lung tumors. By offering enhanced visual clarity, CT scans play a critical role in differentiating between benign and malignant nodules, supporting accurate clinical assessments (8). Additionally, CT imaging serves as a guide for subsequent diagnostic measures, like needle biopsies, and aids in devising appropriate cure strategies and observing disease progression (9). Despite its diagnostic value, the traditional manual interpretation of CT scans poses several challenges. Reviewing a large volume of medical samples is laborious and mentally taxing for experts. This manual process is not only labor-intensive but also prone to inconsistencies, as complex anomalies can go unnoticed or be interpreted differently by different observers. The inherent subjectivity in image analysis eventually results in inter-observer variability, which compromises diagnostic accuracy. Furthermore, with the increasing availability and use of advanced imaging technologies, the sheer amount of data generated has placed an added burden on healthcare professionals. Such limitations demand the requirement for computerized, intelligent systems that can assist in the accurate and efficient interpretation of lung imaging data (10).

Recent advancements in automated lung cancer detection have increasingly relied on imaging data across several modalities, mostly through the application of artificial intelligence (AI) and machine learning (ML) methods. These approaches have appeared as powerful solutions for analyzing complex medical images, which empower them to identify the complicated patterns that are hard to detect through traditional methods (11). AI-driven approaches, especially when applied to CT scans, are capable of efficiently processing huge amounts of imaging data, which supports radiologists in pinpointing minute anomalies associated with lung tumors or nodules. This capability significantly enhances both the speed and precision of diagnosis. By automating the analysis process, AI not only accelerates early detection efforts but also plays a crucial role in standardizing diagnostic practices across diverse clinical environments. It reduces reliance on subjective interpretation, thus minimizing variability between observers and enhancing consistency in results. The integration of intelligent diagnostic systems supports healthcare specialists in making accurate and timely decisions, eventually causing better patient management and improved treatment outcomes (12). These innovations underscore the transformative influence of AI in modern medical imaging and reinforce its value in the fight against lung cancer (13).

Early efforts in lung cancer detection primarily relied on traditional ML algorithms, which involved analyzing hand-crafted features computed from medical samples (14). Investigators focused on quantifiable attributes like the outline, texture, and concentration of suspected lesions in CT scans to perceive and categorize irregularities. These features were then passed to train classifiers that aimed to discriminate between the benign and malignant cases. While this approach yielded encouraging results, it was constrained by the challenges associated with medical image complexity (15). One of the key limitations of traditional ML methods was their dependence on manual feature extraction, which required significant field knowledge and often failed to capture delicate or higher-order patterns within the data. Furthermore, conventional ML techniques struggled to manage the hierarchical and nonlinear characteristics of medical images, which limit their capability to generalize across diverse patient datasets. As a result, their performance in the timely and precise recognition of lung cancer was suboptimal. The advent of DL revolutionized the landscape of medical image examination, mainly in the area of lung cancer classification (16). DL architectures like convolutional neural networks (CNNs) enabled models to automatically compute hierarchical and complicated features right from a given suspected sample (17). Such architectures eradicate the requirements for a manual feature learning procedure and significantly improve the ability to compute fine-grained and abstract patterns associated with cancerous growths (18–20). DL-based models have demonstrated exceptional performance in recognizing sophisticated visual cues, for example, small nodules or early-stage tumors, often undetectable to the human eye. The end-to-end learning capability of deep networks allows them to process raw data and optimize predictions with minimal human intervention, thereby progressing the correctness, consistency, and reliability of lung cancer diagnostics (21–23).

Although DL frameworks have meaningfully advanced lung cancer detection, they are not without limitations. A major concern is the risk of diagnostic errors, including false positives where non-cancerous structures are misidentified as malignant and false negatives, where actual tumors may go undetected. These misclassifications can have serious clinical implications, potentially leading to unnecessary interventions or missed treatment opportunities. Another persistent challenge is the lack of interpretability in DL models. Many DNNs operate as opaque systems, making it problematic for medical experts to understand the reasoning behind a specific prediction, thereby reducing trust and limiting their integration into decision-critical environments. In addition, the computational demands of DL models are substantial. Training and deploying these models typically require high-performance hardware and significant processing time, which can be a barrier to real-time application in clinical workflows, particularly in resource-constrained sites. Furthermore, the outcomes of DL algorithms can be influenced by disparities in imaging protocols, scanner types, and image quality. These inconsistencies across datasets and healthcare institutions may hinder the generalizability of the models, limiting their robustness and effectiveness when applied to new or diverse populations. Addressing these limitations is essential to ensure the safe, efficient, and wide-scale adoption of DL systems in lung tumor recognition and classification.

To address the existing challenges of this field, we propose a refined ResNet50-based model named Residual-SwishNet, designed to enhance classification performance and model robustness. In our approach, the conventional activation functions in the convolutional layers have been replaced with the Swish activation function, known for its smooth, non-monotonic properties that enable improved gradient propagation and feature computation. This modification permits the network to extract complicated patterns more effectually, specifically in complex or low-contrast regions of lung scans. Additionally, we introduce three extra dense layers before the final categorization layer to enrich the feature space and improve the model’s capacity to recognize benign and malignant samples. Further, the usage of the cross-entropy loss method in the softmax layer assists the Residual-SwishNet approach to tackle the class-imbalance problem. These architectural enhancements contribute to more reliable predictions and help mitigate false positives and negatives by offering a more accurate and interpretable solution for lung cancer classification. The main contributions of this framework are listed as:

1. Integration of the Swish activation function in convolutional layers improves non-linear representation learning and model convergence, outperforming traditional functions like ReLU.
2. The addition of three fully connected layers before the classification unit enhances the capability of the approach to extract deep semantic features, which leads to improved decision boundaries.
3. The proposed architecture demonstrates better handling of ambiguous cases due to its high recall ability, which minimizes the incidence of false positives and negatives in lung cancer classification.
4. The model is capable of tackling the class-imbalance issue due to the employment of the cross-entropy loss method in the softmax layer of the proposed approach.
5. The refined model offers a practical and computationally efficient solution that bridges the gap between high accuracy and interpretability, which makes it suitable for deployment in clinical diagnostic workflows.
6. Massive experimental analysis of our work was performed using two standard datasets to demonstrate the efficacy of our method.

The rest of the article comprises the given hierarchy: the related work is provided in Section 2, whereas the suggested work is presented in Section 3. The used dataset, performance indicators, along with a detailed description of achieved outcomes, are given in Section 4. Lastly, the conclusion is stated in Section 5.

## 2 Related work

Here, we have discussed the existing works presented to accomplish the classification task of lung nodules.

Raza et al. (24) proposed a model named Lung-EffNet for classifying lung cancers. Initially, the approach performed data augmentation to increase the sample size. After this, the EfficientNet approach was trained using the concept of transfer learning via applying its variants from B0 to B4. The work was tested over the IQ-OTH/NCCD dataset and obtained a classification score of 99.10%. This work shows good outcomes for categorizing lung nodules; however, it requires a huge number of samples to train the model. Nahiduzzaman et al. (25) introduced a DL method for classifying lung tumors. For this, the model first applied a preprocessing step to enhance the visual representation of the input images. After this, the work applied a model named lightweight parallel depth-wise separable convolutional neural network (LPDCNN) to compute the relevant information of the input images. Next, the Ridge-ELM classifier employed the extracted features from the previous step and performed the categorization of nodules. The work provides better results for binary problem; however, it faces performance degradation for the multiclass classification task. Another DL approach named FocalNeXt was presented in (26) to execute the classification task of lung nodules. The work merged the attention strategy of FocalNet along with the feature computation capability of ConvNeXt inside the vision transformer model. The work has attained a classification score of 99.81%, however, at the cost of an enhanced computing load. Priya et al. (27) proposed a DL model for lung cancer classification. First, samples were preprocessed to improve their appearance. Then, the data size was increased by applying various augmentation techniques. Finally, the work used the SE-ResNeXt-50 approach to calculate the deep features and performed the classification job. The work performed well in recognizing lung cancers; however, it suffered from a high computational cost.

In (28), a DL method was used to recognize the lung tumors. First, a step was followed to eliminate the noisy information from the samples and enhance their visual appearance. The enhanced samples were then passed to the feature extraction phase, where 6 statistical features were estimated based on Improved Empirical Wavelet Transforms (IEWT). After this, the calculated visual characteristics were passed to the Attention-based Convolutional Neural Network with DenseNet-201Transfer Learning (AtCNN-DenseNet-201 TL) to accomplish the categorization task. The approach presents an effective solution to diagnose lung cancer; however, it faces the model overfitting issue. Murthy et al. (29) used an ML approach to categorize lung cancer from CT scans. For this, the Adaptive Median Filter (AMF) was used initially to enhance the quality of the samples. Next, M-SegNet was used to extract the focal areas. After this, the visual information from the segmented regions was computed by computing various types of features named statistical, deep, and textural features, which were combined to make the final vector. After this, the classification was accomplished using the Tree-based Pipeline Optimization Tool with Support Vector Machine (TPOT_SVM). The approach shows better outcomes in the recognition of lung nodules; however, the work is effective for a binary classification task. Mothkura et al. (30) designed a method for classifying lung tumors. The approach used a dense CNN to get dense features and performed the classification task. The work has attained a highest classification rate of 85.21%, which needs further improvements. In (31), a DL network was proposed for lung cancer classification. To accomplish this, the work used s Multimodal Fusion Deep Neural Network (MFDNN) framework that combined features from various types of visual samples like medical imaging, genomics, and clinical data, which was combined for lung cancer classification. The work performed well in recognizing the lung cancer nodules; however, at the cost of a huge computing burden. Mohamed et al. (32) suggested a CNN approach to extract a related group of features and perform the categorization of samples into relevant groups of lung cancers. Further, the work used an optimization strategy to boost the model’s running behavior. The work shows better outcomes in recognizing the relevant cancer groups of the lung; however, the results need further enhancements.

Hossain et al. (33) employed the concept of the ensemble approach for categorizing lung cancer nodules. For this, the approach initially utilized a step to boost the quality of input samples. After this, the work used the CNN-SVD-Ensemble approach to compute the relevant set of visual information and execute the dimensionality reduction. Next, the computed features were passed to various ML classifiers to perform the cataloguing of lung nodules. The strategy gains better outputs in diagnosing the lung tumors; however, it lacks the generalization power. Naseer et al. (34) proposed a DL approach to perform the lung cancer classification task. For this, the approach follows 3 stages, where in the first step, the work used the UNet approach to segment the lobe from CT scans. Next, the enhanced UNet framework was used to compute the nodule mask. In the third stage, the computed masks were passed to a CNN approach named the AlexNet for deep features computation and executing the classification task. The network was tested using the LUNA-16 data sample and attained an accuracy score of 97.98%. The technique attains better scores; however, it requires more improvements. Uddin et al. (35) applied a DL network for lung cancer diagnosis. For this, the work proposed two dense frameworks to learn a representative set of features and execute the recognition task. Further, the work has proposed a hybrid model by joining both networks, and attained a highest classification accuracy of 93%. This technique exhibits better performance in categorizing lung cancer nodules; however, the outcomes need further improvement. In (36), a DL network was designed for the recognition of lung tumors. For this, initially, the samples were preprocessed to enhance the visual information by propagating the sample to the convolution filter and down-sampling it by employing max pooling. Next, the autoencoder approach was applied to compute a set of dense features based on a CNN, and a multispace image reconstruction method was utilized to lessen error while restructuring the sample to enhance the nodule recognition ability of the approach. Lastly, the SoftMax classifier was applied for grouping the samples. Alsallal et al. (37) designed a framework to identify and classify lung tumors. First, various transformations were used to advance the visual aspects of the samples. Next, various structural features were computed by applying the PyRadiomics library. Next, a dense CNN model was merged with attention mechanisms to compute a deep set of sample information. Next, the model used different feature nomination approaches like Non-negative Matrix Factorization (NMF) and Recursive Feature Elimination (RFE). Finally, various ML classifiers like XGBoost and Stacking were applied to perform the classification task. The work performs well for lung cancer classification; however, it needs further improvements.

While numerous studies have explored ML and DL methods for recognizing lung tumor nodules, several limitations remain unresolved. Many existing approaches struggle with issues such as limited generalizability, lack of interpretability, and susceptibility to false predictions. These gaps underline the pressing need for a more effective and robust solution that can enhance feature representation, improve diagnostic accuracy, and ensure consistency across diverse imaging conditions.

## 3 Proposed method

**Figure 1:**
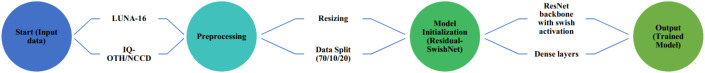
An overview of model training.

**Figure 2:**
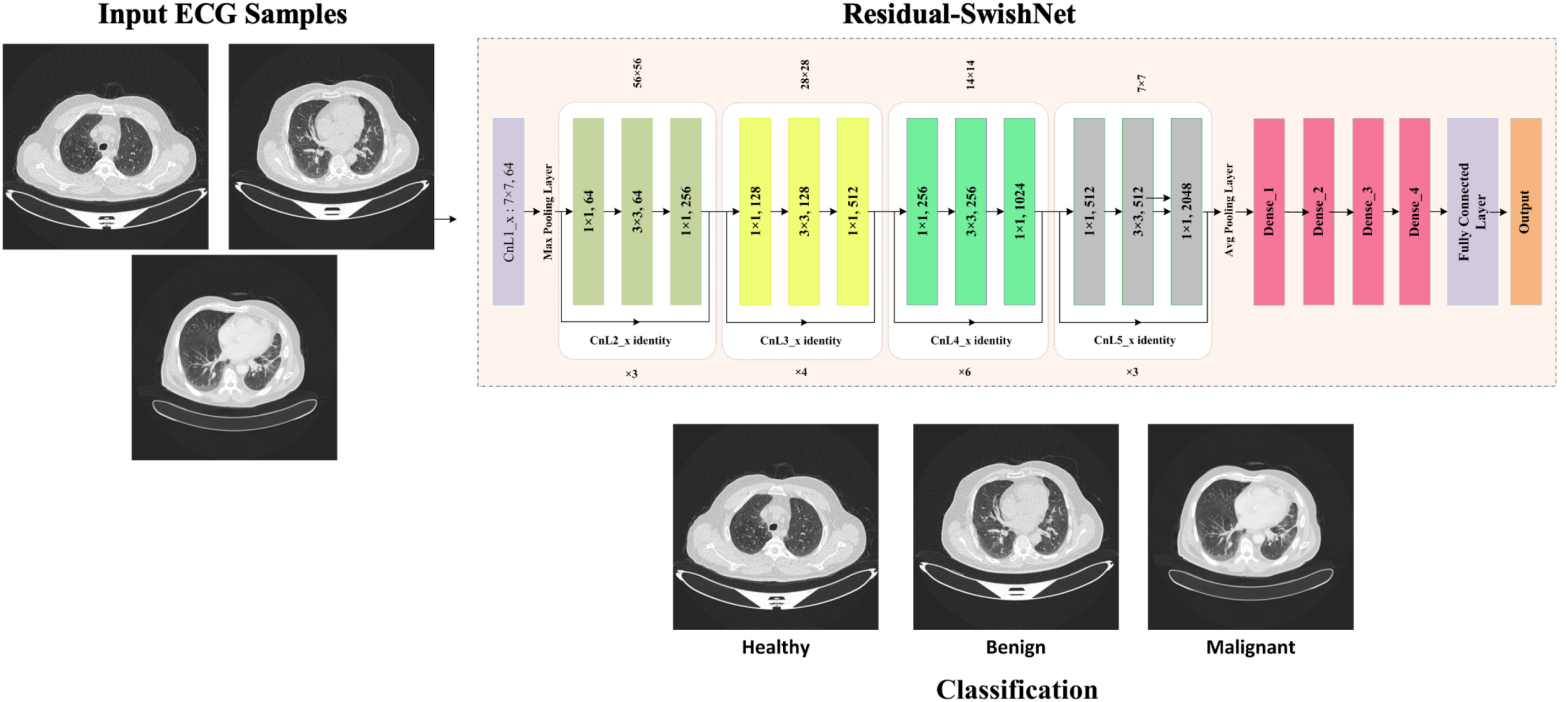
Architectural flow of Residual-SwishNet model.

This study presents a novel DL model named Residual-SwishNet, which is specifically designed to classify lung CT images into benign and malignant groups accurately. The proposed architecture builds upon a refined version of the ResNet50 CNN model, which is modified to capture relevant and complex details found in lung cancer imaging. To improve the learning capability of the approach, we replace the conventional ReLU activation with the Swish activation within the convolutional layers. The smooth and non-monotonic behavior of the Swish activation allows for better gradient flow and the retention of minor negative values, which enhances feature learning in deep layers. Furthermore, the architecture is extended by introducing three additional dense layers before the final classification stage. These layers strengthen the capability of the technique to extract deep semantic features and support more precise decision-making. This enriched feature representation contributes to more reliable classification outcomes, particularly in cases where tumor boundaries or nodular characteristics are complicated. Moreover, to tackle the category imbalance issue, we have used the cross-entropy loss method in the final classification stage. A workflow of the model training is provided in **Figure 1**. For model evaluation, the employed datasets were partitioned into train and test groups, and framework parameters were optimized to ensure stable convergence and robust performance. Residual-SwishNet was then trained to identify key discriminative features in lung CT scans and accurately categorize them into their respective classes. A representation of the designed framework is given in **Figure 2**.

### 3.1 Feature calculation

To boost the accuracy of lung cancer classification, it is essential to compute a robust set of feature representations that effectively capture the differences between malignant and benign lung tissues. High-level features, often referred to as semantic features, are vital in distinguishing such complex visual patterns in CT scan images. In the proposed Residual-SwishNet framework, we utilize a modified ResNet50 backbone to extract these discriminative features. A key enhancement involves replacing the traditional ReLU activation with the Swish, which advances the aptitude of our framework to compute complex spatial information by permitting small negative scores to pass through the network. This helps retain valuable information during feature learning and supports deeper, more expressive representations. As a result, the feature computation stage in Residual-SwishNet becomes more effective in encoding semantic details crucial for improving classification performance.

### 3.2 ResNet50

The base of the proposed Residual-SwishNet framework lies in the ResNet50 architecture (38), which is a powerful and widely adopted deep CNN model known for its use of residual learning. Unlike traditional CNNs that suffer from vanishing gradients as layers deepen, the ResNet50 model addresses this issue through identity-based skip links, which allow gradients to flow more successfully through backpropagation. These skip links empower the system to capture residual mappings rather than direct transformations, which results in both improved convergence and accuracy in deep architectures.

ResNet50 is structured using multiple residual blocks (RBs), each consisting of a series of convolutional layers accompanied by batch normalization and an activation method. The distinguishing feature of an RB is the shortcut path, which allows the input to avoid one or more layers and be directly added to the output of the transformation layers. This process is mathematically represented as Equation 1.

(1) I=R(o)+o

Where *o* is the input to the block, *R(o)* is the residual mapping learned through convolutional operations, and *I* is the final output. This formulation helps hold vital features across layers while enabling the approach to focus on learning only the necessary adjustments. Owing to its architectural efficiency and strong feature extraction capabilities, the ResNet50 model serves as an effective base for the Residual-SwishNet model in analyzing lung CT images for cancer classification.

### 3.3 Residual-SwishNet

**Figure 3:**
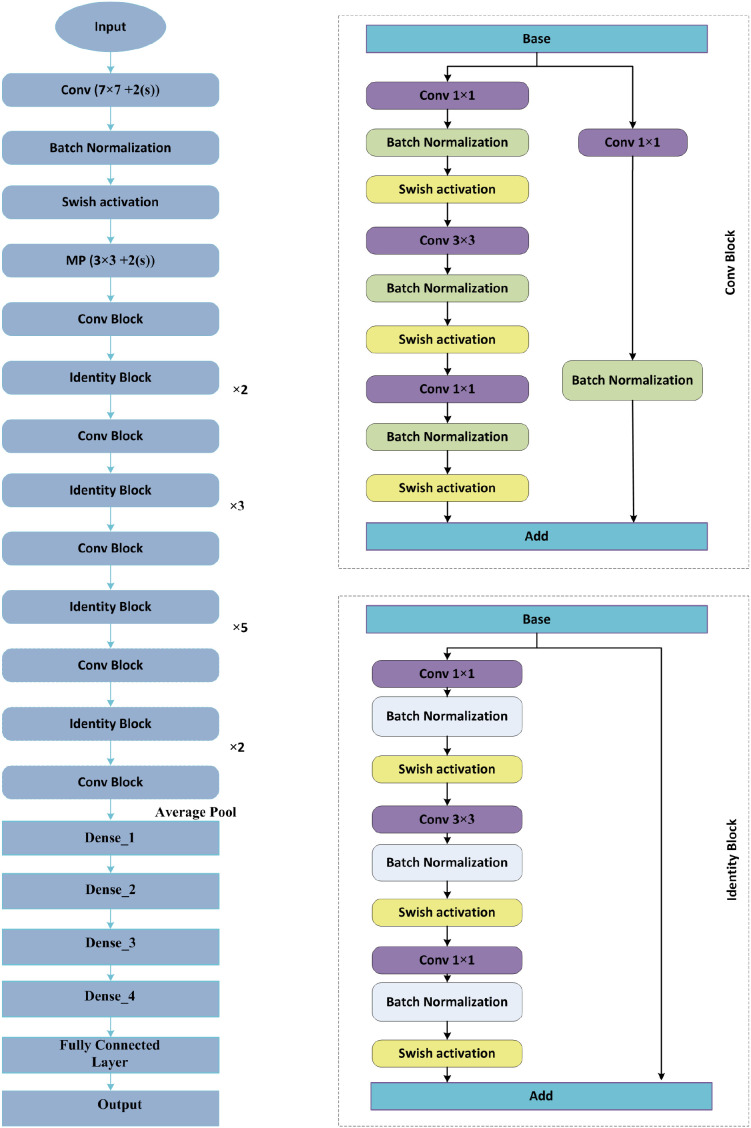
A visual representation of the Residual-SwishNet.

The proposed Residual-SwishNet framework builds upon the ResNet50 architecture (39), which is famous for its residual learning mechanism and robust feature extraction capabilities. Even though ResNet50 performs well in solving various image classification tasks, however comes with certain limitations when applied directly to medical imaging, mainly for detecting complex abnormalities in lung CT scans. Specifically, its dependence on the ReLU activation function leads to the suppression of important negative activations, which can carry useful information in low-contrast medical images. Additionally, the standard architecture is unable to provide sufficient depth in the final layers to fully capture the complicated variations between benign and malignant tissues. To address these challenges, we introduce three key modifications to the original ResNet50 structure, which build the foundation of the Residual-SwishNet model. First, we incorporate the Swish activation function into the residual blocks to replace ReLU in the process of feature capturing. The capability of the Swish method to retain small negative values and promote smoother gradient flow enhances the power of the model to learn complex and refined visual features by improving convergence and reducing training loss. Second, we append 3 extra dense layers before the classification unit to deepen the semantic understanding behavior of the model, which improves its discrimination power before executing the final grouping task. These fully connected layers refine the high-level features and help the model detect more meaningful patterns associated with lung abnormalities. Lastly, we have used the cross-entropy loss technique in the classification layer, which assists the Residual-SwishNet in tackling the sample imbalance problem. Despite these enhancements, the overall complexity of the network remains manageable, which ensures that Residual-SwishNet is suitable for practical clinical applications. The final feature representations are passed to a Softmax layer, which outputs class probabilities for the classification of lung conditions. The architectural overview of Residual-SwishNet is illustrated in **Figure 3**, and a detailed layer-wise structure is provided in **Table 1**. The next sections delve into the internal components of the model in greater detail.

**Table 1:** The layer-wise analysis of Residual-SwishNet.

Layer	Residual-SwishNet
Conv_L1_x	7×7, 64
	3×3 max pooling
Conv_L2_x	$\left[\begin{array}{c} 1\times 1,\;64\\ 3\times 3,\;64\\ 1\times 1,256 \end{array}\right]$× 3
Conv_L3_x	$\left[\begin{array}{c} 1\times 1,\;128\\ 3\times 3,\;128\\ 1\times 1,512 \end{array}\right]$× 4
Conv_L4_x	$\left[\begin{array}{c} 1\times 1,\;256\\ 3\times 3,\;256\\ 1\times 1,1024 \end{array}\right]$× 6
Conv_L5_x	$\left[\begin{array}{c} 1\times 1,\;512\\ 3\times 3,\;512\\ 1\times 1,2048 \end{array}\right]$× 3
Average Pooling	
Dense Layer1	
Dense Layer2	
Dense Layer3	
Softmax layer	

#### 3.3.1 Convolutional layer

In the Residual-SwishNet architecture, convolutional layers form the foundational component responsible for extracting spatially-aware features from lung CT scan images. These layers apply a series of learnable filters to the input image, which enables the technique to detect local patterns such as edges, textures, and shapes that are critical for identifying cancerous regions. As the depth of the network increases, these layers progressively learn more abstract and complex representations, which are essential for distinguishing between benign and malignant conditions. -The operation of a convolutional layer can be mathematically expressed as Equation 2.

(2) $$F_{j}^{n}=f(\sum_{i\in x_{j}}\left(K_{ij}^{n}*P_{i}^{\text{n}-1}+\beta_{j}^{n}\right)$$

Where $F_{j}^{n}$ denotes the output feature map at layer *n*, $\;K_{ij}^{n}$ represents the convolutional kernel applied between input map *i* and output map *j*, ∗ is the convolution operator, $\beta_{j}^{n}$ is the bias term, *f(·)* denotes the activation function (Swish in our case), and $x_{j}$ denotes the total features maps. To standardize the input across all samples and maintain consistency with the architecture, CT scan images are resized to 224 × 224 pixels before being passed through the network. In total, the proposed Residual-SwishNet model incorporates 48 convolutional layers, which are distributed across multiple residual blocks, and each contributes to the hierarchical extraction of features necessary for accurate lung cancer classification.

#### 3.3.2 Activation layer

**Figure 4:**
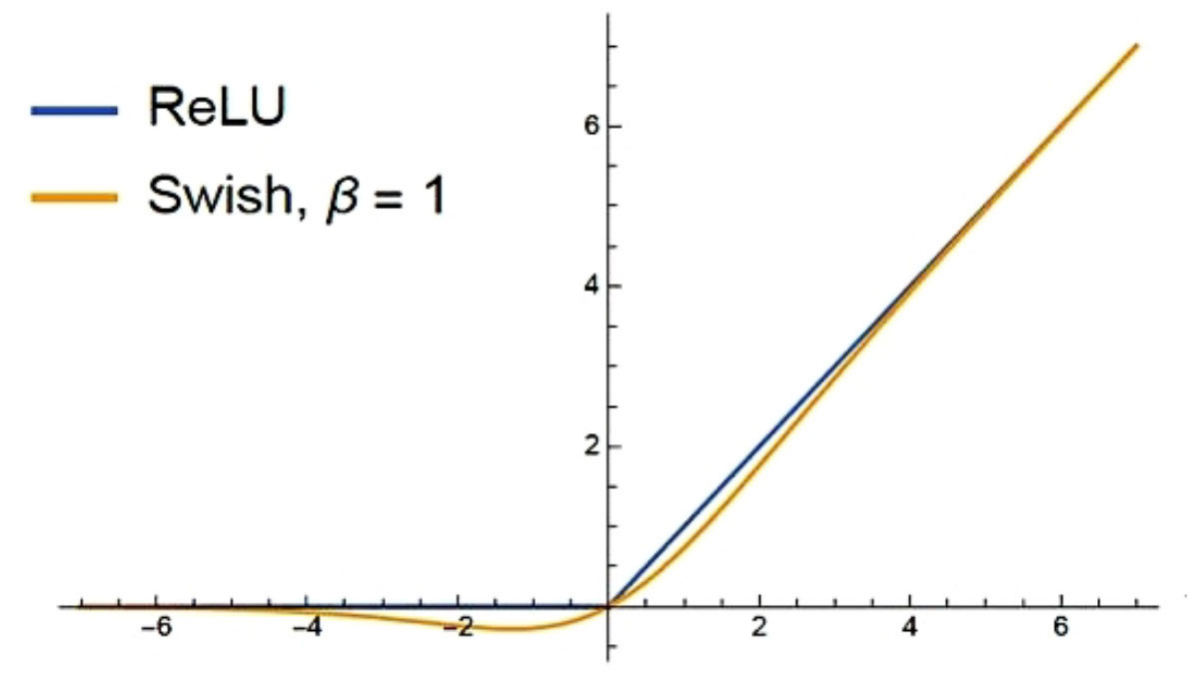
Pictorial analysis of ReLU and Swish activation (40).

To enhance the capacity of the approach to learn fine-grained features from lung CT images, the proposed Residual-SwishNet architecture replaces the conventional ReLU activation with the more advanced Swish activation function after every 2D conv layer. Swish is famous for its smooth and non-linear behavior that enables better gradient propagation and feature retention, particularly in deeper networks. Its ability to pass small negative values in place of completely discarding them, as ReLU does, assists the proposed work in preserving complex patterns in medical images that might otherwise be lost, which is especially critical for detecting early-stage or low-contrast lung abnormalities (40). Unlike ReLU, which is piecewise linear and can lead to dead neuron problems, the employed Swish activation function offers a non-monotonic and differentiable structure, enabling better optimization and more stable training. This leads to stronger generalization performance and improved convergence behavior. A pictorial analysis of the Swish vs ReLU methods is depicted in **Figure 4**. The mathematical expression for the Swish activation is given by Equation 3.

(3) swish(u)=u × σ(ηu)

Where *u* is the input to the activation layer, *σ* denotes the sigmoid function, and *η* is a trainable parameter that scales the input dynamically. However, in our implementation, the parameter η in the Swish equation is fixed to 1 (non-trainable), corresponding to the standard Swish activation rather than the trainable Swish-β variant. The fixed version provided more stable convergence and avoided additional parameters that could lead to overfitting on medical datasets with limited samples. In the context of lung cancer classification, in which learning relevant morphological changes is crucial, the Swish activation approach significantly improves feature sensitivity and learning depth. Its computational efficiency and stability under varying learning rates make it particularly suitable for deep architectures like Residual-SwishNet, which delivers better performance and faster convergence compared to traditional activation functions.

#### 3.3.3 Pooling layer

In the Residual-SwishNet architecture, pooling layers are integrated to reduce the spatial dimensions of feature maps while retaining the most informative patterns. The pooling operation helps in minimizing redundancy, improving computational efficiency, and enhancing the ability of the model to manage spatial variations in lung CT scans by aggregating features from neighboring pixels. Specifically, an average pooling layer is employed before the fully connected stages to summarize the extracted features into a compact vector, which is then passed to the appended dense layers for further processing and classification.

### 3.4 Incorporated dense layers

After the pooling stage, Residual-SwishNet incorporates 3 additional dense layers to refine and enhance the extracted features before classification. The decision to incorporate three dense layers in the final stage of Residual-SwishNet was guided by empirical experimentation conducted during model development. We evaluated multiple configurations, including one, two, three, and more than three dense layers. Models with only one or two dense layers demonstrated weaker feature abstraction and produced lower classification accuracy, indicating insufficient refinement of high-level representations. In contrast, architectures with more than three dense layers began to show signs of overfitting and increased training instability. The configuration with three dense layers consistently achieved the most favorable balance between feature discrimination, computational efficiency, and generalization capability. These layers are activated using ReLU and are focused on emphasizing critical patterns associated with lung abnormalities while filtering out less relevant information. This added depth improves the capacity of the approach to form a more discriminative feature representation from CT scans. The resulting dense feature vector is then forwarded to the Softmax layer for final classification.

### 3.5 Softmax layer

The last stage of the Residual-SwishNet framework utilizes a Softmax layer to convert the dense feature vector into class probabilities, which causes the model to classify lung CT images as benign or malignant. The Softmax function normalizes the outputs of the last dense layer by producing a probability distribution over the target classes. It is defined as Equation 4.

(4) $$\text{σ}(S_{i})=\frac{\text{exp}(S_{i})}{\sum_{j=0}^{n-1}\text{exp}(S_{j})}$$

where $S_{i}$ is the score for class *i*, $S_{j}$ presents the output vectors, and *n* shows the total groups. For optimization, the model uses categorical cross-entropy loss (41), which measures the divergence between predicted and true class labels and supports learning even under class imbalance.

## 4 Results

In this section, a thorough explanation of the employed datasets, along with the evaluation parameters, is provided. Further, a vast evaluation of the Residual-SwishNet is performed to prove the robustness of our network.

### 4.1 Implementation details

**Figure 5:**
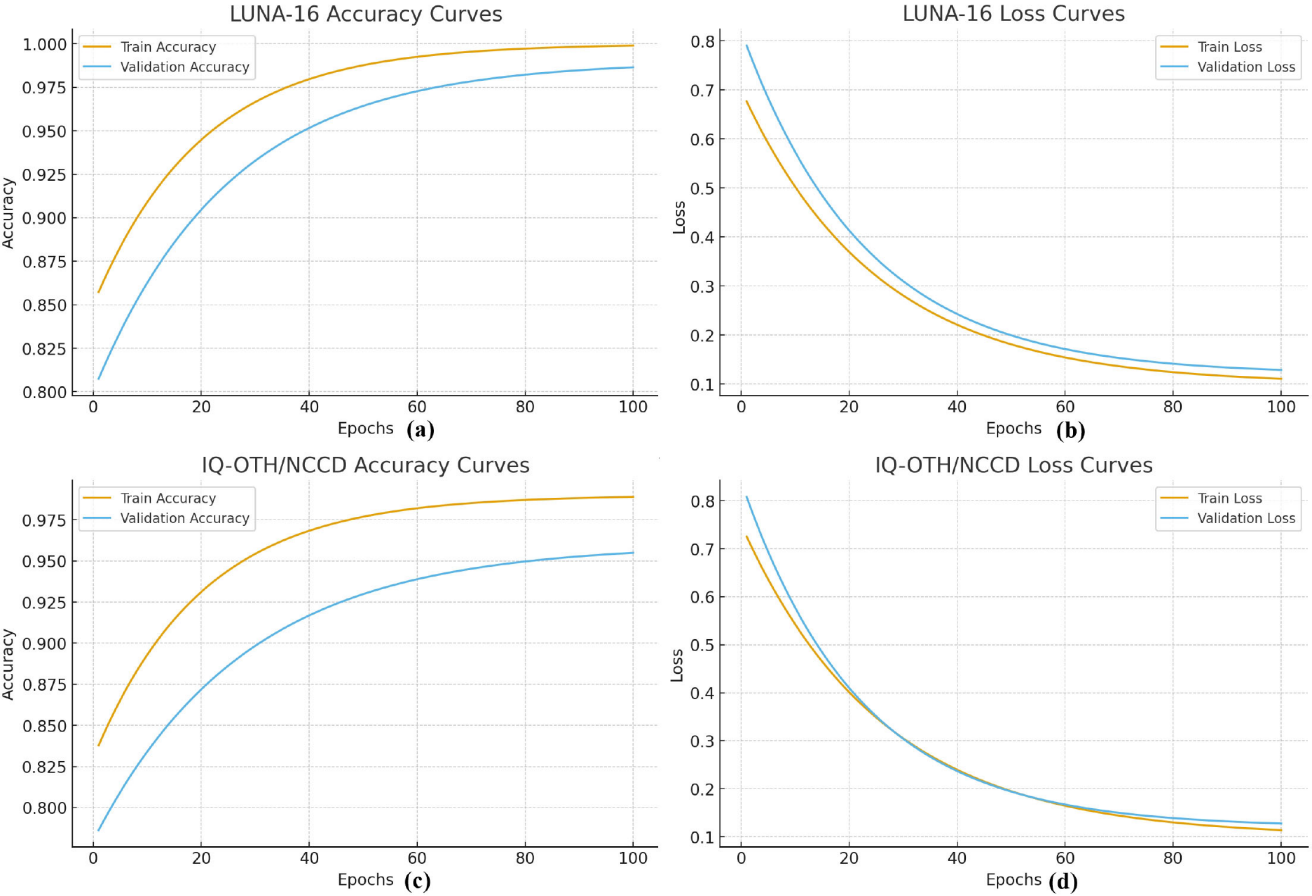
LUNA-16 Training curves **(a)**, LUNA-16 Loss curves **(b)**, IQ-OTH/NCCD Training curves **(c)**, IQ-OTH/NCCD Loss Curves **(d)**, respectively.

The proposed Residual-SwishNet was implemented using Python and TensorFlow/Keras. The model was optimized using the Adam optimizer with an initial learning rate of 0.0001, chosen for stable convergence when training deep residual networks. A batch size of 32 was used for all experiments to maintain a balance between training stability and computational efficiency. Both datasets were divided into 70% training, 10% validation, and 20% testing splits to enable fair performance evaluation. The model was trained for 100 epochs with early stopping based on validation loss. Further, **Figure 5** shows the training and validation curves for both datasets. As the epochs increase, the accuracy for both training and validation steadily improves, while the loss gradually decreases. The two curves stay close to each other, which means the model is learning well and not overfitting. These curves confirm that our Residual-SwishNet trains smoothly and generalizes well to unseen data.

### 4.2 Evaluation parameters

To measure the classification outcomes of the proposed Residual-SwishNet technique, we have used the standard evaluation parameters named accuracy, precision, recall, and F1-Score, which are computed as mentioned in Equations 5–8.

(5) $$Accuracy=\frac{TP+TN}{TP+FP+TN+FN}$$

(6) $$\text{Precision}=\frac{TP}{TP+\;FP}$$

(7) $$\text{Recall}=\frac{TP}{TP+\;FN}$$

(8) F1−Score=(2×Precision×Recall)(Precision+Recall)

### 4.3 Dataset

To train and test the proposed approach, we utilized two standard online repositories of lung cancer samples, namely LUNA-16 (42) and IQ-OTH/NCCD lung cancer dataset (43), both of which were accessed on July 01, 2025. The LUNA16 dataset, a curated subset of the publicly available LIDC-IDRI repository, is a broadly recognized standard for tuning networks for lung cancer diagnosis. It contains chest CT scans that have been meticulously annotated by expert radiologists, who evaluated each scan to confirm the incidence, locality, and characteristics of pulmonary nodules. As part of its refinement, the LUNA16 dataset excludes CT images with slice thicknesses greater than 2.5 mm to ensure consistent imaging quality across the dataset. The complete dataset comprises 888 CT scans, which are reviewed by four radiologists, providing reliable annotations of the size, location, shape, and density of nodules. These images focus specifically on the lung regions containing nodules, allowing the framework to extract appropriate patterns. To facilitate the lung tumors classification task, the nodules were divided into two categories based on the mean malignancy score assigned by radiologists. Nodules with a mean score of 2.5 or below were categorized as benign, indicating non-cancerous conditions, while those with a mean score of 3.5 or higher were classified as malignant, reflecting a higher likelihood of cancer. The second data sample utilized in this work is the IQ-OTH/NCCD lung cancer dataset, which was gathered at the National Center for Cancer Diseases in Iraq over a period of 3 months during the fall of 2019. This sample comprises a total of 1,190 CT image slices obtained from 110 victims and delivers an adequate amount of data for framework tuning. The dataset is organized into three distinct categories: normal, benign, and malignant cases. Among the collected samples, there are 416 images labeled as normal, 120 as benign, and 561 as malignant, which indicates a minor imbalance in class distribution. The major reason to choose these datasets is due to their complex natures and presence of various image distortions, which make them challenging for accomplishing the lung cancer classification task.

### 4.4 Model evaluation

This section is focused on discussing the output results of the Residual-SwishNet approach by indicating the attained scores on two employed standard datasets named LUNA16 and IQ-OTH/NCCD lung cancer data samples, with the employment of various measures like precision, recall, F1, and Accuracy metrics.

**Figure 6:**
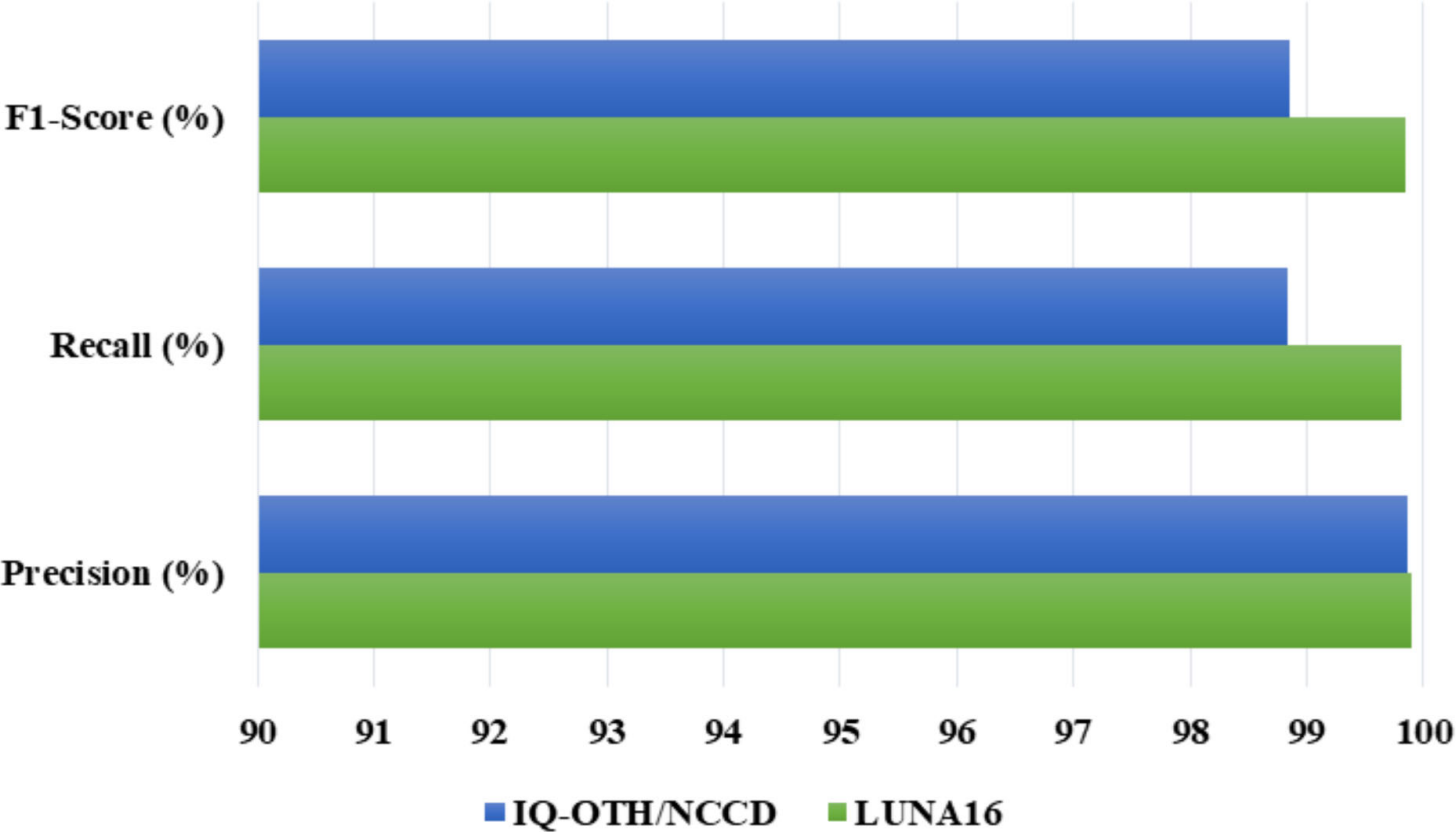
Residual-SwishNet results over the LUNA16 and IQ-OTH/NCCD datasets using precision, recall, and F1-score measures.

To thoroughly assess the results of our proposed Residual-SwishNet model, first, we discussed the classification metrics named precision, recall, and F1-score on both the LUNA16 and IQ-OTH/NCCD samples, and attained analysis is provided in **Figure 6**. These metrics offer a complete understanding of the diagnostic strength and reliability of our model, which is vital in health imaging tasks where both false positives and negatives can possess serious clinical consequences. Precision reflects the capability of an approach to appropriately recognize true positive cases among all predicted positives, which is vital to minimizing unnecessary interventions. Recall, on the other hand, computes the capacity of the work to detect all actual positive cases by ensuring that no malignant or suspicious case is overlooked. Lastly, the F1-score presents a harmonic mean of precision and recall, which balances these two aspects by delivering a single robust indicator of classification performance, especially under class-imbalanced conditions common in medical datasets. Using line bar visualizations, we plotted these metrics across both datasets to showcase how Residual-SwishNet consistently achieves high scores across all three metrics, showing the efficacy of our network. These outcomes confirm that our approach is competent to maintain high diagnostic accuracy while reducing both false alarms and missed detections, proving its effectiveness in real-world clinical screening circumstances.

**Figure 7:**
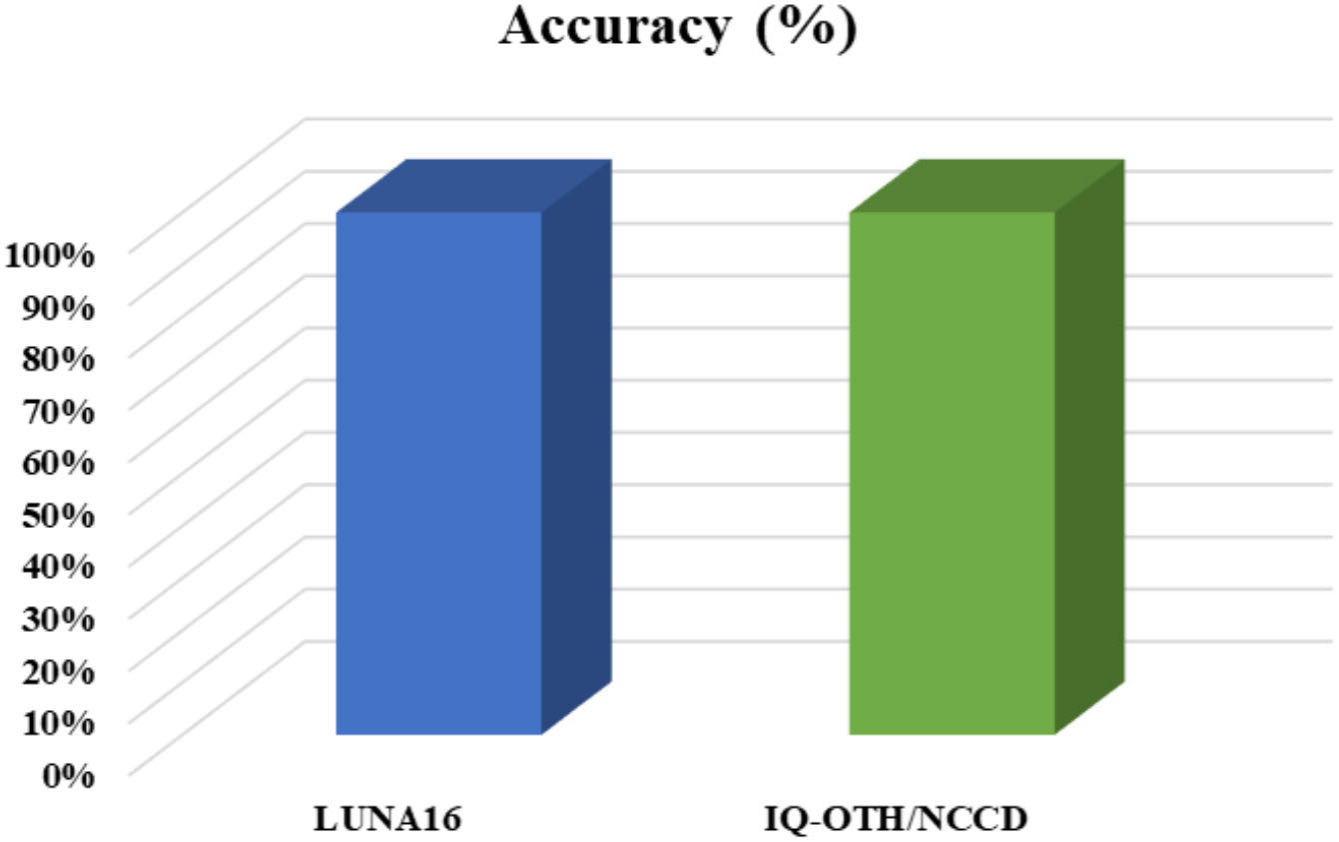
The Residual-SwishNet results representation over the LUNA16 and IQ-OTH/NCCD datasets using accuracy metrics.

In addition to evaluating precision, recall, and F1-score, we also analyzed the accuracy of our proposed model across both datasets, and the results are shown in **Figure 7**. Accuracy is one of the most fundamental metrics in classification, which computes the proportion of correctly predicted instances over the total number of predictions. On both the LUNA16 and IQ-OTH/NCCD datasets, our model consistently outperformed and attained improved accuracy values. It can be seen from the values provided in **Figure 7** that our approach offers comprehensive capability to correctly classify a wide range of lung cancer cases, regardless of their complexity or similarity across classes. The improved accuracy is due to the better information-capturing capability of the approach, which ensures minimal misclassification and boosts the model’s reliability for clinical use.

**Figure 8:**
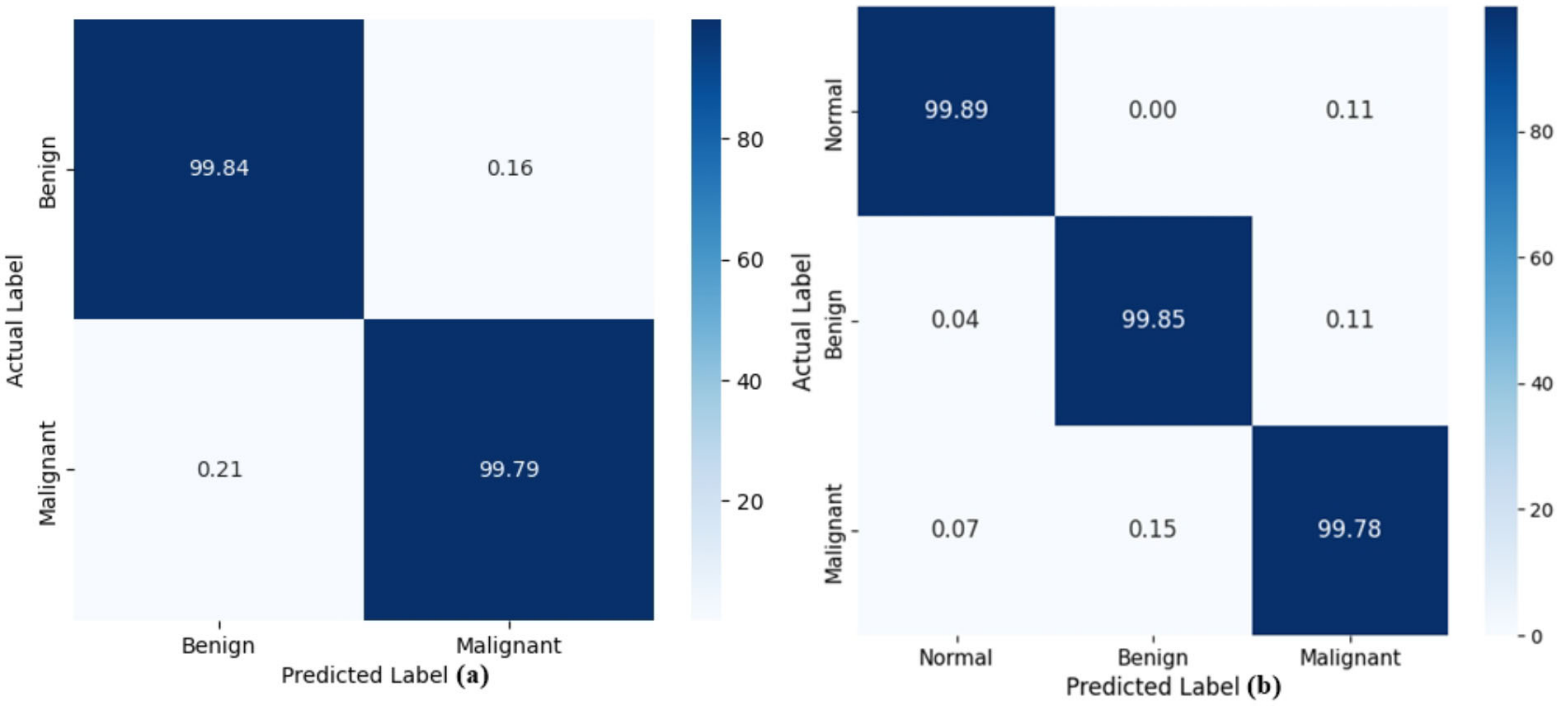
Confusion matrix attained by the Residual-SwishNet over **(a)** LUNA16, **(b)** IQ-OTH/NCCD, respectively.

Next, we have discussed the confusion matrix for both employed datasets, and visuals are provided in **Figure 8**. The confusion matrices shown in **Figure 8** not only reflect the exceptional performance of the proposed model on the LUNA16 and IQ-OTH/NCCD datasets but also serve as a critical tool for evaluating classification effectiveness. A confusion matrix provides a comprehensive view of the predictive capabilities of an approach by presenting true positives, true negatives, false positives, and false negatives in a structured format. This aids in analyzing explicit zones where the approach surpasses or needs development, beyond what single-value metrics like accuracy or F1-score can convey. In addition to the visual confusion matrices shown in **Figure 8**, we also provide the numerical confusion matrix values in tabular form to improve clarity in **Tables 2**, **3** for LUNA-16 and IQ-OTH/NCCD datasets, respectively. These tables present the exact number of correctly and incorrectly classified samples for each class in both datasets, enabling a more precise interpretation of the model’s performance. Specifically, for the LUNA16 dataset, the model accurately classifies 99.84% of benign and 99.79% of malignant cases, with extremely low misclassification rates of 0.16% and 0.21% respectively, indicating high discriminative power between the two classes. While for the IQ-OTH/NCCD dataset, presenting a 3-class problem named normal, benign, and malignant, the confusion matrix again demonstrates excellent performance with 99.89%, 99.85%, and 99.78% correctly predicted for each class, respectively. The extremely low off-diagonal values confirm the capability of the approach to avoid confusion between clinically similar categories. These strong results validate the effectiveness of the proposed modifications, like replacing ReLU with Swish activation, incorporating three dense layers, and using a Softmax output layer with Cross-Entropy Loss, in enhancing feature learning and decision boundaries, ultimately leading to superior classification precision and reliability.

**Table 2:** Tabular representation of the Confusion matrix for LUNA16 dataset.

Actual/predicted	Benign	Malignant
	TPR (%)	
Benign	99.84	0.16
Malignant	0.21	99.79

**Table 3:** Tabular representation of the Confusion matrix for IQ-OTH/NCCD dataset.

Actual/Predicted	Normal	Benign	Malignant
	TPR (%)		
Normal	99.89	0.00	0.11
Benign	0.04	99.85	0.11
Malignant	0.07	0.15	99.78

**Figure 9:**
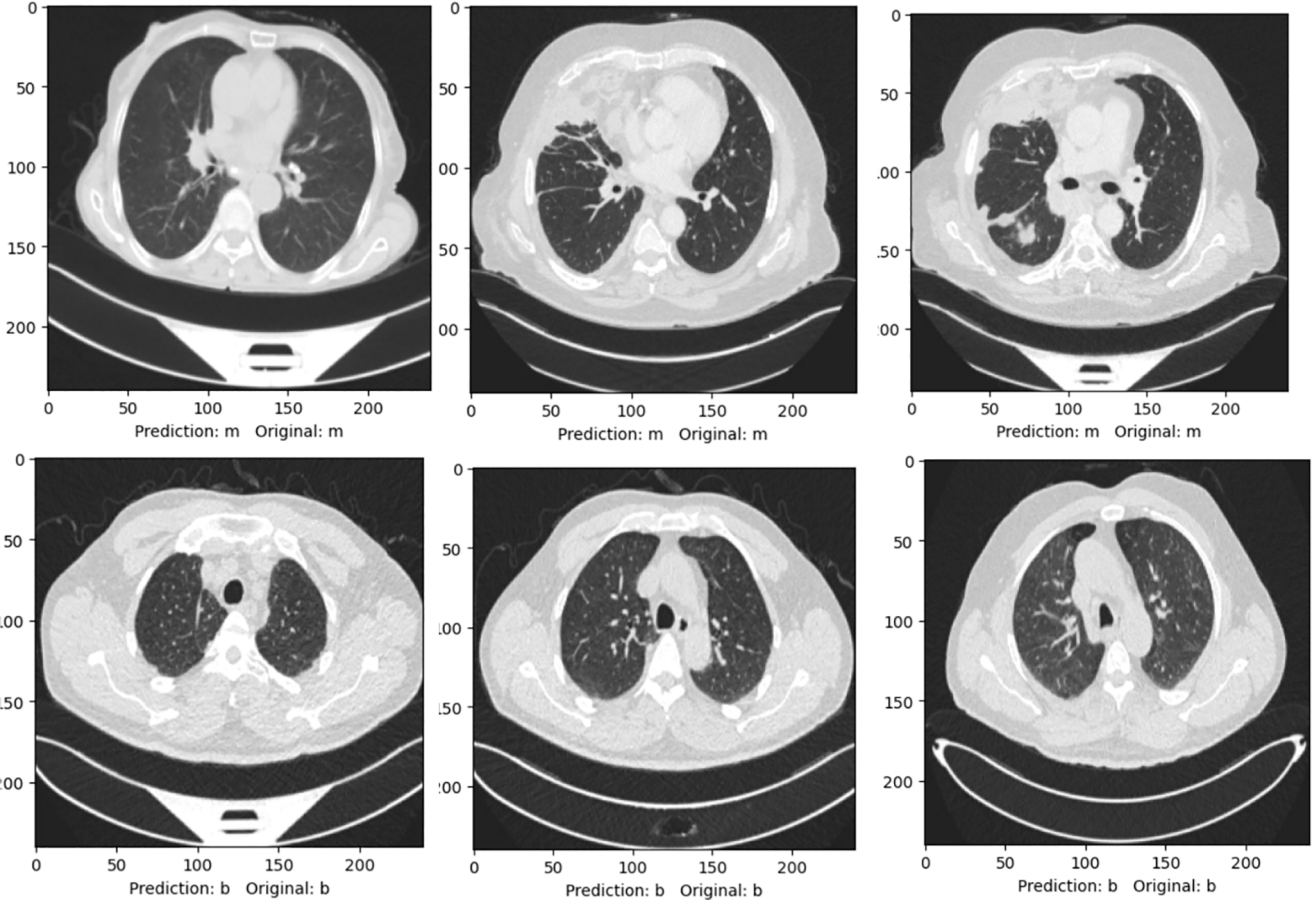
A visual representation of actual and predicted labels by the Residual-SwishNet.

To qualitatively assess the model’s recognition capability, we presented a few example CT scan images marked with both the predicted and original labels in **Figure 9**. It can be seen from the visuals given in **Figure 9** that the Residual-SwishNet accurately classifies the cases, clearly demonstrating its ability to generalize and correctly identify critical patterns in complex lung imagery. Such visual validations strengthen the confidence in our proposed solution by highlighting its potential reliability in clinical environments. The sharp correspondence between the projected and real labels confirms the robustness of the proposed architecture across diverse imaging conditions.

**Figure 10:**
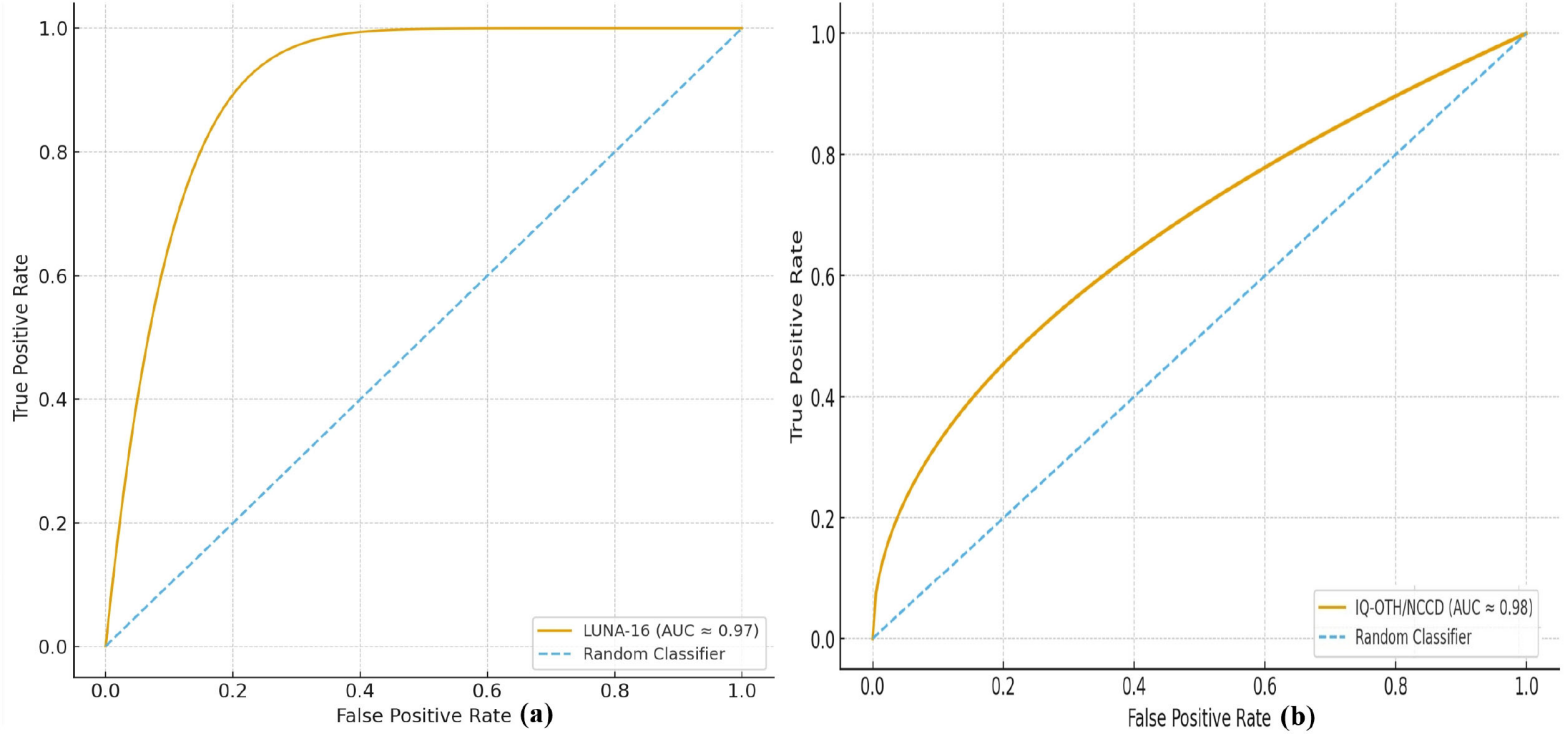
ROC curves for both datasets **(a)** LUNA-16, **(b)** IQ-OTH/NCCD using the Residual-SwishNet approach.

To further validate the robustness of Residual-SwishNet, ROC curves were generated for both datasets. As shown in **Figure 10**, the model achieves an AUC of approximately 0.97 on LUNA-16 and 0.98 on IQ-OTH/NCCD, indicating excellent separability between benign and malignant cases across both datasets. The curves rise sharply toward the top-left corner, demonstrating a high true-positive rate with minimal false positives, which confirms stable generalization beyond accuracy and F1-score results. These ROC curves provide additional evidence that the proposed method maintains strong discriminative performance and does not rely on chance-level decision boundaries.

**Figure 11:**
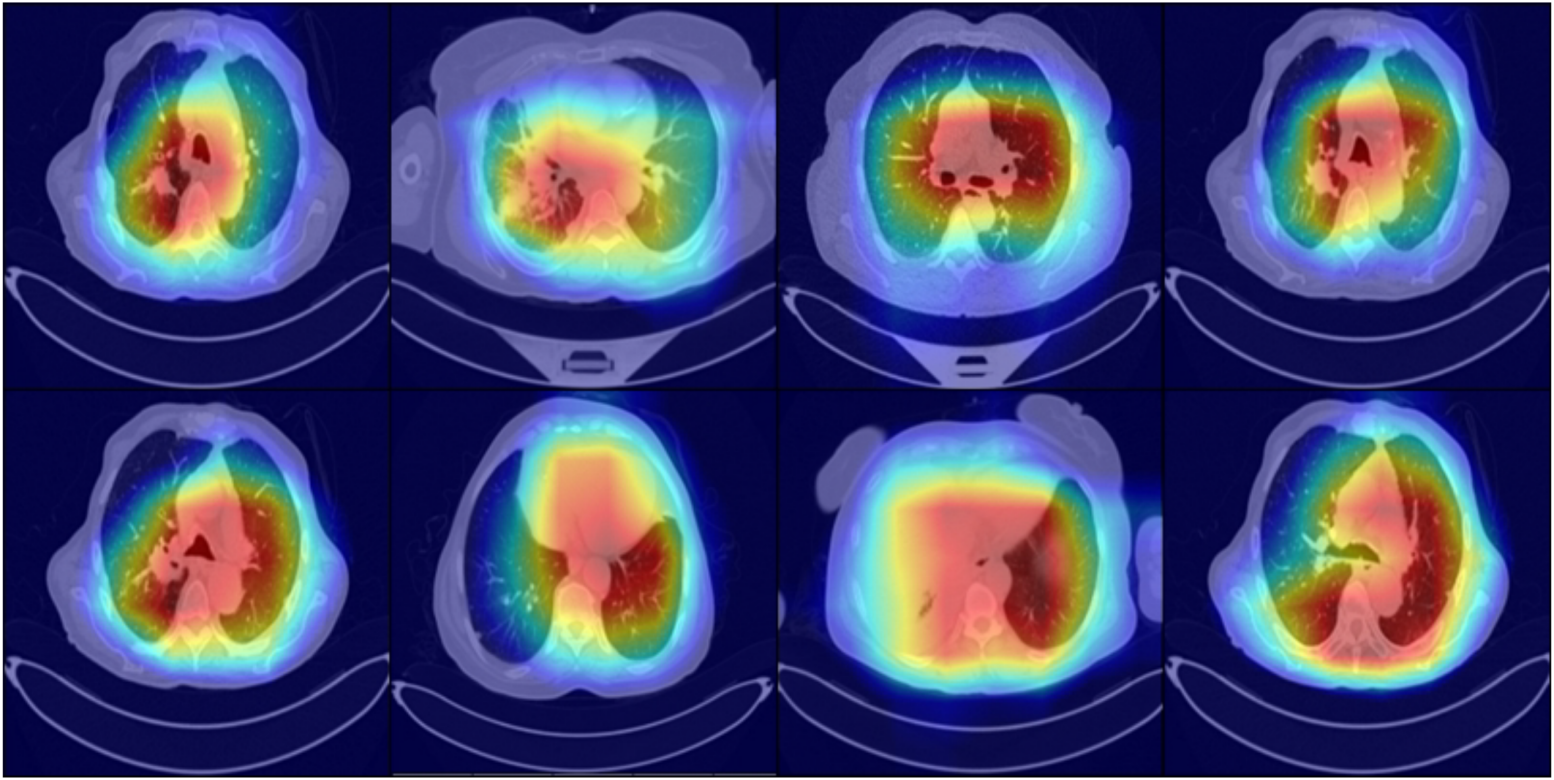
Heatmap analysis of the proposed approach for lung cancer classification.

To further support the interpretability of the proposed model, we have generated heatmaps using Grad-CAM, and attained visuals are given in **Figure 11**. These visualizations highlight the explicit areas within the CT scans that the Residual-SwishNet emphasizes when making predictions. The activation regions primarily concentrate on the interior portions of the lungs, indicating that the model is correctly attending to clinically relevant areas for feature extraction. This not only validates the internal decision-making process of the deep learning model but also aligns with expert radiological understanding, thus reinforcing the clinical reliability and transparency of the system.

So, after providing a comprehensive assessment of the Residual-SwishNet approach using multiple performance metrics, including precision, recall, F1-score, and accuracy, along with visual analysis through confusion matrices, predicted label overlays, and Grad-CAM heatmaps, we have confirmed the robustness of our approach for lung cancer classification. The consistently high scores across both datasets and the strong qualitative evidence highlight the effectiveness, robustness, and interpretability of the model in classifying lung cancer cases. These evaluations confirm that the model not only attains advanced performance but also offers transparency in its predictions, which is an essential requirement for real-world medical applications.

### 4.5 Comparison with DL approaches

In this part, we have analyzed the performance of the Residual-SwishNet approach in comparison to numerous well-known DL approaches for both employed datasets, named LUNA16 and IQ-OTH/NCCD lung cancer data samples.

First, the scores for the LUNA16 dataset are discussed by using the standard evaluation measures, namely precision, recall, F1-score, and accuracy, and the obtained analysis is shown in **Table 4**. For evaluation, various DL approaches, i.e., the EfficientNet series (44), NASNetMobile (45), DenseNet121 (46), MobileNetV2 (47), and MobileNet (48), are taken as provided in (49). The assessment in **Table 4** clearly displays that the proposed solution ranked highest in all evaluation measures with scores of 99.90%, 99.81%, 99.85%, and 99.60% for the precision, recall, F1-Score, and accuracy metrics. Further, the EfficientNetB2 model attains comparable performance with scores of 95.50%, 94.60%, 95.10%, and 95.40% against the precision, recall, F1-score, and accuracy measures. Among all DL models, the NASNetMobile approach shows the worst performance in classifying the lung cancer samples with scores of 88.25%, 86.65%, 87.65%, and 87.45% over the precision, recall, F1, and accuracy metrics. The proposed solution shows the best solution due to its high recognition power. On the LUNA16 dataset, the comparative DL models show better results; however, these models are subject to certain limitations in the context of lung cancer classification. The EfficientNet and EfficientNetV2 series techniques present optimized solutions for parameter efficiency; however, at the cost of compromised accuracy due to their shortcoming in capturing the complex textural differences present in lung nodules, as these models employ lightweight convolutional layers and standard activation functions. Such a structure of the EfficientNet family limits their feature sensitivity. Further, NASNetMobile and MobileNet architectures prioritize computational efficiency for mobile applications but sacrifice depth and feature extraction capabilities, which also makes them less suitable for high-detail medical imaging tasks. DenseNet121, though effective in feature reuse, lacks specialized modifications tailored to lung cancer detection and does not optimally capture domain-specific patterns. In contrast, our Residual-SwishNet enhances feature learning through a ResNet50 backbone equipped with the Swish activation function, which improves non-linear learning and gradient flow, and integrates additional dense layers to refine feature representation. These improvements enable Residual-SwishNet to better capture fine-grained nodule characteristics, resulting in superior classification accuracy, sensitivity, and specificity compared to these general-purpose models.

**Table 4:** Residual-SwishNet comparative analysis with DL models for the LUNA16 dataset.

Framework	Precision	Recall	F1-Score	Accuracy
EfficientNet-B0	95.60%	93.50%	95%	95.30%
EfficientNet-B1	95.70%	94.40%	94.90%	95.20%
EfficientNet-B2	95.50%	94.60%	95.10%	95.40%
EfficientNet-B3	95.30%	94.60%	94.70%	95.40%
EfficientNet-V2-B0	95.55%	94.45%	94.85%	95.25%
EfficientNet-V2-B1	95.65%	94.35%	94.75%	95.15%
EfficientNet-V2-B2	95.45%	94.55%	95.35%	94.95%
NASNetMobile	88.25%	86.65%	87.65%	87.45%
DenseNet-121	86.80%	89.10%	88.50%	87.50%
MobileNet-V2	87.30%	88.80%	87.90%	89.00%
MobileNet	87.90%	88.50%	87.40%	88.80%
**Proposed**	**99.90%**	**99.81%**	**99.85%**	**99.60%**

Bold values mean result attained by our model.

Next, the comparison results for the proposed solution are discussed over the IQ-OTH/NCCD dataset using various DL models like DenseNet-121 (46), Inception-V3 (50), MobileNet-V2 (47), ResNet-50 (51), ResNet-152 (52), ResNet101 (53), as given in (54, 55), and the acquired evaluation is given in **Table 5**. Again, the results show that our model, named Residual-SwishNet, performs better than the selected DL approaches in classifying lung cancer over the IQ-OTH/NCCD dataset with a precision of 99.86%, along with F1-score and accuracy values of 98.85% and 99.11%. The comparative models have proven effective in generic image classification tasks; however, they exhibit key limitations in medical imaging contexts. DenseNet-121 and Inception-V3, despite their depth and feature reuse capabilities, rely on conventional activation functions and lack targeted optimization for lung cancer detection. MobileNet-V2 is a lightweight architecture that compromises feature depth for computational efficiency, which reduces its ability to extract detailed lung nodule patterns. Even the deeper ResNet variants are powerful, but employing ReLU activation and standard architectural setups, which limit their capacity to capture detailed variations critical in lung CT scans. In contrast, our Residual-SwishNet builds on ResNet50 but overcomes these limitations through the integration of the Swish activation, which advances gradient propagation and feature sensitivity. Further, introducing additional dense layers resulted in a more refined feature representation. These enhancements allow Residual-SwishNet to extract and process intricate features more effectively, resulting in improved results across all key measures on the IQ-OTH/NCCD dataset.

**Table 5:** Residual-SwishNet comparative analysis with DL models for the IQ-OTH/NCCD dataset.

Framework	Precision	Recall	F1-Score	Accuracy
DenseNet-121	95.50%	–	95.50%	95.50%
Inception-V3	96.30%	–	95.50%	95.50%
MobileNet-V2	95.90%	–	95.80%	95.80%
ResNet-50	92.82%	92.22%	92.83%	94.18%
ResNet-152	87.90%	–	87.40%	87.40%
ResNet101	93.26%	86.63%	86.97%	94.43%
**Proposed**	**99.86%**	**98.84%**	**98.85%**	**99.11%**

Bold values mean result attained by our model.

### 4.6 Comparison with the state-of-the-art

In this part, we have evaluated the designed work against various latest approaches employing the same dataset to validate its robustness in classifying the lung cancer nodules.

First, we have discussed evaluation results for the LUNA16 dataset by analyzing its outcomes with numerous new works (27, 49, 56–60). Priya et al. (27) suggested a DL framework named SE-ResNeXt-50 to classify the lung cancer nodules and attained an accuracy score of 99.15%. In (60), a DCSwinB dual-branch DL method was proposed that combines CNN-based local feature extraction with Swin Transformer-based global context modeling, fused through a Conv-MLP module for enhanced 3D representation, and attained an accuracy score of 90.96%. The approach in (49) proposed a CNN approach with an attention strategy to recognize lung nodules from CT-Scan images. The CNN unit computed deep features on which an attention module was employed to recognize the relevant information. The work reported an accuracy score of 95.40%. Thangavel et al. (56) utilized a DL model to categorize pulmonary nodules from suspected images. Initially, some preprocessing steps were performed to enhance the visual quality of samples, on which the TNet DL model was applied to segment the focal regions from the CT-Scan images. After this, the CenterNet technique was applied to compute visual aspects from the extracted clusters. At last, the NASNet approach was applied to execute the classification task. This dense network has obtained an accuracy score of 99.29%. Next, discussed work in (57) proposed a model named LungNet-SVM for lung cancer classification. The approach presented an improved AlexNet approach for deep features computation, on which the classification was carried out by the SVM classifier. The work has achieved an accuracy score of 97.64%. Next, the method in (58) applied Gabor filters along with an improved Deep Belief Network (E-DBN) to compute the visual information from the given images. Further, for the classification task, the approach used numerous classifiers, with the highest value attained by the SVM classifier with an accuracy of 99.161%. Alsheikhy et al. (59) used the VGG-19 model with long short-term memory networks (LSTMs) to execute the classification task of lung cancer nodules. This technique has stated an accuracy value of 99.42%. The results depicted in **Table 6** indicate that the Residual-SwishNet approach has attained the highest results in terms of all evaluation parameters used in the assessment compared to all the modern approaches. We have reported an accuracy score of 99.60% with a performance gain of 2.31%. Further, in terms of precision, the proposed model has reported a performance gain of 3.98%, which is 3.21% and 3.18% for the recall and F1-Score, respectively.

**Table 6:** Residual-SwishNet comparative analysis with new works for the LUNA16 dataset.

References	Year	Precision	Recall	F1-Score	Accuracy
(27)	2025	99.15%	97.58%	98.54%	99.15%
(60)	2025	85.56%	90.56%	90.56%	90.96%
(49)	2024	95.80%	94.69%	95.24%	95.40%
(56)	2024	99.19%	99.22%	99.20%	99.29%
(57)	2023	–	96.37%	–	97.64%
(58)	2023	–	98.048%	–	99.161%
(59)	2023	99.88%	99.76%	99.82%	99.42%
**Proposed**	**2025**	**99.90%**	**99.81%**	**99.85%**	**99.60%**

Bold values mean result attained by our model.

The main cause of attaining effective scores in comparison to the latest approaches is because of the robust information learning capability of the suggested solution. The work in (27) improved features recalibration but lacked advanced activation mechanisms to compute complex lung patterns. Further, the work in (60) lacks to handle the distorted samples. Similarly, a CNN with attention in (49) enhanced focus on key regions but was limited by the shallow feature extraction backbone. Further, the approach in (56) followed a multi-stage pipeline combining which increased complexity and risked cumulative errors. Earlier models, like improved AlexNet with SVM (57) and Gabor filters combined with Deep Belief Networks (E-DBN) and SVM (58), relied on outdated or manually crafted features, restricting their depth and adaptability. Furthermore, the method in (59) computed dense features; however, it suffered from the model overfitting issue. In contrast, our proposed Residual-SwishNet overcomes these limitations through an optimized ResNet50 backbone integrated with the Swish activation function, enhancing nonlinear learning and preserving critical negative activations. Additionally, we introduced three dense layers for richer feature abstraction, creating a streamlined, end-to-end framework that efficiently learns from lung CT images and outperforms prior methods in results and robustness on the LUNA16 dataset.

Next, we have carried out the comparative analysis of the Residual-SwishNet for the IQ-OTH/NCCD dataset against the latest approaches (61–65), and the obtained values are provided in **Table 7**.

**Table 7:** Residual-SwishNet comparative analysis with new works for the IQ-OTH/NCCD dataset.

References	Year	Precision	Recall	F1-Score	Accuracy
(61)	2025	99.84%	97%	99.79%	99.06%
(62)	2025	99.06%	98.82%	98.94%	99.00%
(63)	2024	90.01%	91.78%	90.88%	89.36%
(64)	2024	99.45%	98.20%	98.82%	97.32%
(65)	2024	98.70%	97.50%	98.24%	98.82%
**Proposed**	**2025**	**99.86%**	**98.84%**	**98.85%**	**99.11%**

Bold values mean result attained by our model.

Kumar et al. (61) suggested a DL approach for lung cancer classification, where VGG19 was used to compute deep features, while for the classification, the Vision Transformer (ViT) was applied. This work attained an accuracy of 99.06%. The work in (62) presented a hybrid approach employing various DL methods like GoogLeNet, EfficientNet, DarkNet19, and ResNet18 to perform the diagnosis of lung tumors, and stated an accuracy of 99%. Venkatraman et al. (63) also designed a DL framework for lung nodules classification, in which VGG16 was used to compute deep features, while for the classification task, the SVM approach was used. The work reported an accuracy value of 89.36%. Further, the work in (64) employed an improved GoogLeNet approach with Adaptive Layers named GoogLeNet-AL for recognizing lung cancer nodules. The technique has acquired a categorization score of 97.32%. Gupta et al. (65) proposed a DL method for lung cancer classification that presented an enhanced U-Net framework in which a conventional U-Net model was used for multi-scale features computation along with the Differentiable Architecture Search. The work achieved an accuracy of 98.82%. In comparison, the suggested framework again attained the highest values for all measures over the IQ-OTH/NCCD dataset in comparison to all approaches. The improved feature engineering capability, along with the high recall rate of the proposed approach, assists the model in attaining robust results. On the IQ-OTH/NCCD dataset, prior studies revealed several architectural and methodological limitations. For instance, the work in (61) combined VGG19 with ViT, but the shallow feature extraction of VGG19 and the data-hungry nature of ViT limited performance on smaller medical datasets. Similarly, ensemble models in (62) introduced unnecessary computational overhead without guaranteeing significant performance gains. Other works, like those discussed in (63, 64), were constrained by outdated or shallow backbones that struggled to extract the complicated visual patterns of lung cancer. Segmentation-based models like those discussed in (65) added additional pipeline complexity, which was not optimal for direct classification tasks. In contrast, our Residual-SwishNet effectively integrates a deep ResNet50 backbone with Swish activation and added dense layers, enabling rich feature learning without excessive complexity. Further, the inclusion of cross-entropy loss in the final classification layer helps to tackle the class-imbalance problem. The overall architectural description of the DeepLungNe assists in computing fine-grained patterns by minimizing training inefficiencies and leads to superior classification results across key performance metrics.

### 4.7 Cross-dataset evaluation

To evaluate the generalization capability of the proposed model, we performed cross-dataset experiments using the benign and malignant classes from two different datasets: LUNA-16 and IQ-OTH/NCCD, and attained results are provided in **Table 8**. In the first setting, the model was trained on the LUNA-16 dataset and tested on IQ-OTH/NCCD, and attained an accuracy of 65.41%. In the second setting, the model was trained on IQ-OTH/NCCD and tested on LUNA-16, resulting in an accuracy of 59.93%.

**Table 8:** Cross-dataset evaluation of the Residual-SwishNet approach.

Training Dataset	Testing Dataset	Accuracy (%)
LUNA-16	IQ-OTH/NCCD	65.41
IQ-OTH/NCCD	LUNA-16	59.93

These results show that the model retains a reasonable level of recognition ability even when tested on entirely unseen datasets, which shows that the learned features by the proposed approach possess a degree of transferability. The better performance when trained on LUNA-16 is attributed to its larger size and greater variability, which allow the Residual-SwishNet to learn richer and more generalizable representations. However, the drop in accuracy in both cases highlights the challenges posed by differences in image acquisition protocols, resolution, and noise characteristics between datasets. Although the cross-dataset results are promising, there is still room for improvement. Incorporating advanced domain adaptation methods, more robust data augmentation strategies, or transfer learning fine-tuning could help bridge the performance gap and make the model more resilient to domain shifts, ultimately enhancing its real-world applicability.

## 5 Conclusion

This study has introduced a DL approach named Residual-SwishNet for lung cancer classification. Specifically, we altered the ResNet50 framework by integrating the Swish activation function, additional dense layers, and a Softmax output with Cross-Entropy Loss to enhance learning capacity and classification precision. The work is evaluated on two standard datasets, named the LUNA16 and IQ-OTH/NCCD datasets, and achieved accuracy scores of 99.60% and 99.11%, outperforming existing state-of-the-art methods. These results highlight the competency of our approach to accurately differentiate between benign and malignant lung nodules by offering potential support in early diagnosis. However, one limitation of our approach is the increased computational complexity and training time introduced by model modifications, which can limit deployment on resource-constrained devices or real-time applications. The proposed Residual-SwishNet carries strong clinical relevance, as accurate early differentiation between benign and malignant lung nodules can support radiologists and reduce diagnostic delays. However, the study has certain limitations, including the use of only two publicly available datasets, which may introduce dataset-specific biases and limit generalizability. External validation on multi-center clinical data and more diverse patient populations is still required. Additionally, although the cross-dataset evaluation shows promising transferability, the performance gap highlights the need for further robustness improvements. Future research will focus on integrating domain adaptation techniques, incorporating 3D volumetric information, and validating the model in real clinical workflows to enhance reliability and practical adoption.

## Data Availability

Publicly available datasets were analyzed in this study. This data can be found here: https://www.kaggle.com/datasets/avc0706/luna16, https://www.kaggle.com/datasets/hamdallak/the-iqothnccd-lung-cancer-dataset.
